# MSRLNet: A Multi-Source Fusion and Feedback Network for EEG Feature Recognition in ADHD

**DOI:** 10.3390/brainsci15111132

**Published:** 2025-10-22

**Authors:** Qiulei Han, Ze Song, Hongbiao Ye, Yan Sun, Jian Zhao, Lijuan Shi, Zhejun Kuang

**Affiliations:** 1 College of Computer Science and Technology, Changchun University, Changchun 130022, China; 231502538@mails.ccu.edu.cn (Z.S.); 241501498@mails.ccu.edu.cn (H.Y.); 231501502@mails.ccu.edu.cn (Y.S.); zhaojian@ccu.edu.cn (J.Z.); kuangzhejun@ccu.edu.cn (Z.K.); 2 Key Laboratory of Intelligent Rehabilitation and Barrier-Free for the Disabled (Ministry of Education), Changchun University, Changchun 130022, China; shilj@ccu.edu.cn; 3 Jilin Provincial Key Laboratory of Human Health Status Identification & Function Enhancement, Changchun 130022, China; 4 Jilin Rehabilitation Equipment and Technology Engineering Research Center for the Disabled, Changchun 130022, China; 5 College of Electronic Information Engineering, Changchun University, Changchun 130022, China

**Keywords:** attention deficit hyperactivity disorder, EEG microstates, CNN–GRU, feature fusion, adaptive feedback optimization, data augmentation, small-sample learning

## Abstract

Background: Electroencephalography (EEG) has been widely used in Attention Deficit Hyperactivity Disorder (ADHD) recognition, but existing methods still suffer from limitations in dynamic modeling, small-sample adaptability, and training stability. This study proposes a Multi-Source Fusion and Feedback Network (MSRLNet) to enhance EEG-based ADHD recognition. Methods: MSRLNet comprises three modules: (1) Multi-Source Feature Fusion (MSFF), combining microstate and statistical features to improve interpretability; (2) a CNN-GRU Parallel Module (CGPM) for multi-scale temporal modeling; and (3) Performance Feedback–driven Parameter Optimization (PFPO) to enhance training stability. Feature-level data augmentation is introduced to alleviate overfitting in small-sample scenarios. Results: On a public dataset, MSRLNet achieved an accuracy of 98.90%, an F1-score of 98.98%, and a kappa of 0.979, all exceeding comparative approaches. Conclusions: MSRLNet shows high accuracy and robustness in ADHD EEG feature recognition, verifying its potential application value in clinical auxiliary diagnosis.

## 1. Introduction

Attention Deficit Hyperactivity Disorder (ADHD) is a common neurodevelopmental disorder, characterized by persistent inattention, hyperactivity, and impulsivity, which severely affect children’s learning, behavior, and social functioning [1]. In recent years, EEG has been widely applied to the objective analysis and recognition of ADHD, providing important evidence for exploring its neural mechanisms and supporting clinical diagnosis [2].

However, traditional EEG methods for ADHD mainly focus on static extraction of time-, frequency-, and statistical-domain features, such as power spectral density or Hjorth parameters [3,4]. These approaches often fail to capture dynamic abnormalities of brain function, particularly the deep temporal and spatial dynamics of EEG signals. Moreover, certain traditional indices, such as the theta/beta ratio, lack sufficient stability for reliable use in ADHD diagnosis, limiting further improvement of existing classification models. Although deep learning models have achieved promising results in EEG-based diagnosis, most of them behave as black boxes with limited neurophysiological interpretability, which hinders their acceptance in clinical decision-making.

In recent years, deep learning (DL) has shown strong capability in automatic feature extraction and modeling for EEG decoding tasks [5,6]. Convolutional neural networks (CNN) are effective in capturing local spatial features [7], while recurrent networks such as GRU and LSTM excel in temporal modeling.

Nevertheless, most existing DL models still face challenges in ADHD feature extraction: public EEG datasets for ADHD include few subjects, making sample size insufficient to support deep learning, leading to overfitting in small-sample training and reduced generalization.

Second, the lack of effective characterization of dynamic brain changes limits the modeling of complex EEG rhythms. For example, most MI-EEG classification models directly use raw EEG as network input, which avoids manual feature design but fails to capture more complex EEG dynamics [8]. García-Ponsoda et al. [9] extracted handcrafted statistical features from segmented EEG for ADHD classification, confirming the influence of preprocessing and temporal resolution on performance, but their method also remained confined to static features and could not model the temporal evolution of brain activity.

Moreover, clinical EEG data are often affected by non-stationarity [10] and noise [9]. Static training parameters cannot adapt to performance fluctuations across stages, and the lack of effective adaptive mechanisms hinders clinical application.

EEG microstate analysis has emerged as an important tool for characterizing dynamic brain changes. It has been widely applied in neuropsychiatric research in recent years [11]. A microstate refers to a brief period (about 60–120 ms) during which scalp potentials maintain a stable topographic distribution, thought to reflect synchronized activation of large-scale neural networks with a clear neurophysiological basis [12].

Microstate features have shown discriminative power in the recognition of schizophrenia [13,14,15], Alzheimer’s disease [16,17,18], and depression [19,20,21], and provide good spatiotemporal sensitivity and interpretability in identifying neural states such as cognitive load and arousal. However, their application in ADHD recognition remains at an early stage, with existing studies lacking effective integration of statistical features and the ability to model non-stationary structures. Integrating microstate features with statistical descriptors provides a complementary view of brain activity: microstates capture fast-changing neural dynamics with clear topographic meaning, while statistical features summarize long-term signal stability and individual variability. This dual-level fusion not only enriches feature representation but also enhances interpretability, offering a bridge between data-driven modeling and clinically meaningful EEG biomarkers—an advantage that recent single-modality or purely deep-learning approaches have not fully realized.

In summary, existing methods remain limited by insufficient feature fusion, restricted dynamic modeling, and poor adaptability to non-stationarity. To address these issues, we propose a Multi-Source Fusion and Feedback Network (MSRLNet) with the following innovations:

1. Feature level: EEG microstate features and multidimensional statistical features are fused at the input layer for the first time, integrating static and dynamic information to enhance neurophysiological interpretability.
2. Modeling level: A CNN-GRU parallel structure is designed to combine local convolution with global temporal modeling, enabling efficient capture of multi-scale dynamic dependencies.
3. Optimization level: A performance feedback-driven adaptive mechanism is introduced, together with feature-level data augmentation, significantly improving stability and generalization under small-sample conditions.

Together, these designs form an ADHD EEG feature recognition framework that achieves sufficient feature fusion, structural robustness, and clinical potential. Unlike previous models that rely solely on deep networks or handcrafted features, the proposed fusion of EEG microstates and statistical features integrates neurophysiological interpretability with computational efficiency. This framework thus represents a step toward clinically reliable and explainable EEG-based ADHD diagnosis.

## 2. Materials and Methods

### 2.1. Datasets

The experimental data of this study were obtained from the public dataset “EEG data for ADHD/Control children” [22]. The dataset includes 121 children aged 7–12 years (61 ADHD, 60 healthy controls), with both genders represented. ADHD subjects were diagnosed by psychiatrists according to DSM-IV criteria, while controls had no history of psychiatric or neurological disorders. EEG was recorded following the international 10–20 system [23] with 19 channels (Fz, Cz, Pz, C3, T3, C4, T4, Fp1, Fp2, F3, F4, F7, F8, P3, P4, T5, T6, O1, O2), at a sampling rate of 128 Hz, referenced to the earlobes.

During data collection, participants performed a visual attention task in which they counted cartoon characters displayed on a screen. This task-based paradigm was designed to elicit attention-related neural activity, providing richer temporal–spatial EEG dynamics than resting-state conditions.

All EEG signals underwent standardized preprocessing, including band-pass filtering, artifact removal, and independent component analysis, ensuring data quality and reliability of neural sources.

### 2.2. Data Preprocessing

To enhance feature extraction and model training, a standardized EEG preprocessing pipeline was established, including band-pass filtering, artifact correction, re-referencing, and independent component analysis (ICA). This process effectively removed various non-neural interferences while preserving neural information.

Raw EEG was first band-pass filtered at 0.5–45 Hz to retain δ, θ, α, β, and low-γ rhythms while eliminating ocular, muscular, and power-line artifacts. High-amplitude segments were then automatically corrected using the ASR method with thresholds (0.25, 0.75) to improve stability. These thresholds were determined based on the statistical energy distribution of EEG signals and the balance between artifact suppression and neural information preservation. The lower limit facilitates the detection of high-amplitude artifacts, whereas the upper limit prevents excessive correction, thereby maintaining signal fidelity and ensuring stable preprocessing. Based on artifact correction, average re-referencing was applied to reduce bias and enhance ICA separation accuracy. Finally, ICA was performed, and ICLabel was used to identify brain-related components; only components with confidence >0.7 were retained for signal reconstruction.

**Figure 1:**
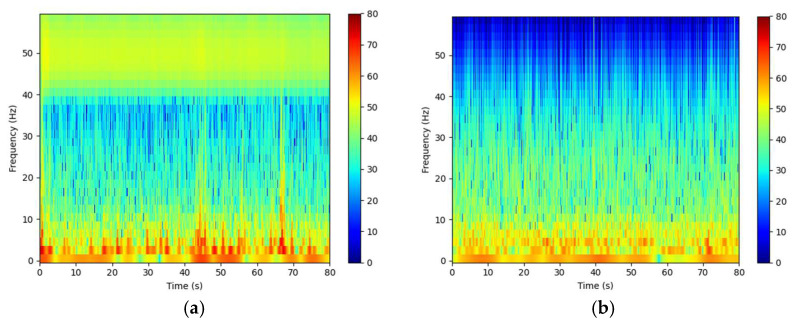
Time–frequency representations from a randomly selected participant. (**a**) Time–frequency plot of raw EEG data. (**b**) Time–frequency plot after preprocessing.

This pipeline not only removed artifacts but also improved the time–frequency structure of EEG, providing higher-quality input for subsequent feature extraction and model development, as shown in Figure 1.

### 2.3. Feature Extraction

#### 2.3.1. Statistical Feature Extraction

**Figure 2:**
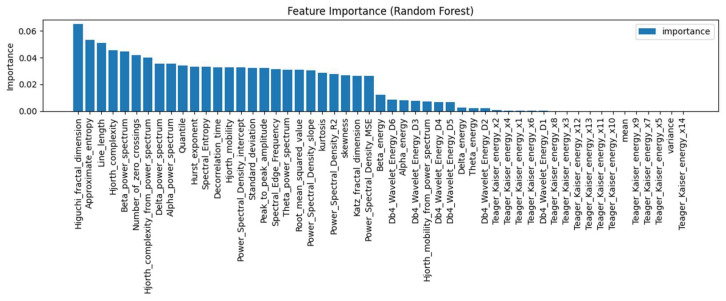
Features used in [9].

To comprehensively capture the time–frequency and nonlinear characteristics of ADHD EEG signals, we adopted the framework of García-Ponsoda et al. [9] and extracted 53 statistical features across 26 categories using the mne-features library (Figure 2), covering time-, frequency-, and nonlinear domains.

**Figure 3:**
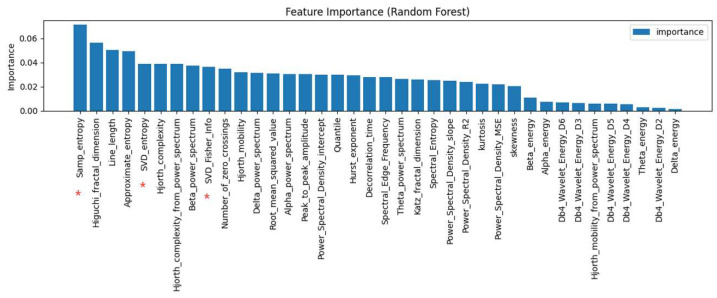
Features adopted in this study (“*” indicates the newly added feature).

To enhance discriminability and training efficiency, Random Forest–based feature selection was applied. Features with a Gini index below 0.002 were discarded, yielding 37 high-contribution features across 23 categories (Figure 3).

In addition, considering the complexity and non-stationarity of ADHD EEG signals, three information-theoretic features were further introduced: Sample Entropy, SVD Entropy, and SVD-based Fisher Information, to enhance the characterization of signal nonlinearity and structural uncertainty. These features showed high importance in ranking (marked with “*” in Figure 3), effectively supporting the discrimination of ADHD EEG patterns. Their definitions are as follows:

(1) Sample Entropy: Measures the variation in signal self-similarity across embedding dimensions.

(1) $$SampEn\left(m,r\right)=-\ln(\frac{A}{B}),\;A=\sum_{i=1}^{N-m}C_{i}^{m+1}\left(r\right),B=\sum_{i=1}^{N-m}C_{i}^{m}\left(r\right)$$

where $C_{i}^{m}\left(r\right)$ denotes the number of sequences similar to the i-th subsequence under embedding dimension m and tolerance r.

(2) SVD Entropy: Quantifies the entropy of the singular value distribution of the reconstructed signal.

(2) $$SVDEn=-\sum_{i=1}^{k}p_{i}\ln p_{i},{\;p}_{i}=\frac{\sigma_{i}}{\sum_{j=1}^{k}\sigma_{j}}$$

where $\sigma_{i}$ is the i-th singular value from SVD, and k is the dimension threshold retaining the main energy.

(3) SVD-based Fisher Information: Evaluates local variation and orderliness of the singular value distribution.

(3) $$\text{SVD-FI}=\sum_{i=2}^{k}\frac{{\left(p_{i}-p_{i-1}\right)}^{2}}{p_{i}+\varepsilon },\;\;\;\varepsilon =10^{-8}$$

where $p_{i}$ is defined as above, and ε is a small constant preventing division by zero (set to $10^{-8}$ here).

In total, the constructed feature set comprised 40 dimensions, integrating statistical, spectral, and nonlinear dynamical information, thereby providing a solid basis for the efficient training and accurate discrimination of the MSRLNet model (Table 1).

#### 2.3.2. Microstate Feature Extraction

To characterize the short-term stable topographic structures of EEG signals, this study extracts microstate-based dynamic evolution features. The overall workflow comprises GFP peak detection, template clustering alignment, sequence labeling, and feature computation.

First, the GFP at each time point is calculated to identify representative frames, defined as:

(4) $$GFP\left(t\right)=\sqrt{\frac{1}{N}\sum_{i=1}^{N}{\left(V_{i}\left(t\right)-\bar{V}\left(t\right)\right)}^{2}}$$

where $V_{i}\left(t\right)$ denotes the potential of channel i at time t, $\bar{V}\left(t\right)$ is the mean potential across electrodes, and N is the number of channels. Higher GFP values indicate stronger spatial differentiation, typically corresponding to stable topographies.

Subsequently, GFP peak frames were extracted for K=4 clustering to obtain individual templates. After aggregation, a second clustering was applied to generate global templates. To standardize the numbering, the reference templates of Koenig et al. [37] were adopted, and template alignment was performed by maximal correlation matching:

(5) $$\hat{T_{k}}=\arg\underset{j}{\max}\left|\mathrm{corr}\left(C_{k},T_{j}\right)\right|$$

All microstates were then uniformly labeled as classes A–B–C–D.

After obtaining the microstate label sequence, the following features were extracted:

1. Mean Duration: The mean duration of each state is defined as:

(6) $$Duration_{k}=\frac{1}{n_{k}}\sum_{i=1}^{n_{k}}d_{i}$$

where $n_{k}$ is the number of occurrences of state k, and $d_{i}$ is the duration of the i-th occurrence.

2. Time Coverage: The proportion of each state within the entire EEG segment is defined as:

(7) $$Coverage_{k}\;=\;\frac{T_{k}}{T}$$

where $T_{k}$ is the total time of state k, and T is the overall duration.

3. State transition probability: A 4×4 transition matrix was constructed. After removing diagonal self-transitions, 12 transition probabilities were retained as features.

(8) $$P_{i,j}=\frac{N_{i\to j}}{\sum_{k}N_{i\to k}}$$

where $N_{i\to j}$ denotes the number of transitions from state i to state j.

4. Global Explained Variance (GEV): Reflects the fitting ability of templates to EEG spatial distributions, defined as:

(9) $$\mathrm{GE}V_{k}=\frac{\sum_{t\in S_{k}}{\mathrm{corr}}^{2}\left(V_{t},T_{k}\right)\cdot \mathrm{GFP}{\left(t\right)}^{2}}{\sum_{t}\mathrm{GFP}{\left(t\right)}^{2}}$$

Finally, each EEG segment was transformed into a unified feature vector, including microstate temporal statistics (12D), transition probabilities (12D), and GEV features (4D).

This feature set was used as one of the inputs to the MSRLNet model, together with statistical features, to support ADHD feature recognition.

### 2.4. Methods

**Figure 4:**
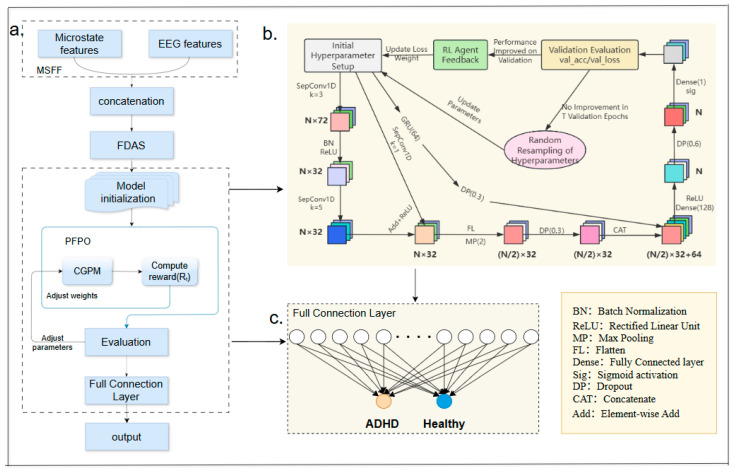
Schematic of the MSRLNet model. (**a**) Overall workflow; (**b**) Performance feedback parameter tuning; (**c**) Fully connected classification layer.

To effectively model the complex dynamics and multi-source information in ADHD EEG signals, we propose a multi-source fusion feedback-regulated network, MSRLNet. The overall architecture is shown in Figure 4a and consists of three functional modules and one training strategy: (1) Multi-Source Feature Fusion mechanism (MSFF); (2) CNN–GRU Parallel Modeling structure (CGPM); (3) Performance Feedback-driven Parameter Optimization mechanism (PFPO); (4) Feature-level Data Augmentation strategy (FDAS).

#### 2.4.1. Multi-Source Feature Fusion Mechanism

MSFF integrates microstate features (see Section 2.3.2) and statistical features (see Table 1) into a unified representation, capturing both dynamic and static EEG characteristics. Features from different sources are normalized and reference-corrected across time, channel, and scale dimensions to ensure compatibility and consistency in feature space. Subsequently, all features are concatenated into a unified multi-source input vector:

(10) $$X_{\mathrm{fused}}=\left[X_{\mathrm{micro}},X_{\mathrm{stat}}\right]$$

By jointly embedding microstate-derived temporal–topographic descriptors and statistical indicators of signal stability, MSFF enables a complementary integration of transient neural dynamics and global EEG regularities. This joint representation not only bridges the gap between low-level temporal patterns and high-level statistical summaries but also enhances feature discriminability and neurophysiological interpretability. Compared with conventional single-source representations, the proposed fusion captures richer multi-scale dependencies, thereby improving the model’s generalization to individual variability in EEG patterns and providing a compact yet informative basis for robust CGPM modeling.

#### 2.4.2. CNN–GRU Parallel Modeling Structure

The overall CGPM structure consists of a convolutional branch (CNN) and a temporal branch (GRU), responsible for extracting local convolutional features and global temporal features, respectively. The two feature streams are then fused and fed into the classifier for ADHD discrimination of input samples.

Let the fused input feature be denoted as matrix $X\in R^{T\times D}$, where T is the number of time steps and D the feature dimension at each step. The convolutional branch first applies two SeparableConv1D layers with different kernel sizes to extract short- and mid-term temporal features:

(11) $$H_{1}=\mathrm{ReLU}\left(BN\left({\mathrm{SepConv}}_{3}\left(X\right)\right)\right),\;{\;\;\;\;\;H}_{2}=\mathrm{BN}\left({\mathrm{SepConv}}_{5}\left(H_{1}\right)\right)$$

To enhance feature integration, a residual connection is introduced: the input X is compressed by a 1 × 1 convolution to obtain $H_{res}$, which is added to $H_{2}$ and activated to produce the final convolutional output:

(12) $$H_{res}={\mathrm{SepConv}1D}_{k=1}\left(X\right),\;H_{cnn}=\mathrm{ReLU}\;\left(H_{2}+H_{\mathrm{res}}\right)$$

Subsequently, $H_{cnn}$ undergoes max pooling, flattening, and Dropout to yield the local convolutional representation:

(13) $$F_{cnn}=\mathrm{Dropout}(\mathrm{Flatten}(\mathrm{MaxPool}1D(H_{cnn})))$$

Max pooling was adopted for its efficiency and robustness to noise compared with other pooling strategies [38].

The GRU branch directly takes X as input, modeling long-term dependencies through gated mechanisms. GRU was employed for its compact structure and stable convergence, which have proven effective in EEG-based CNN–GRU models [39].

(14) $$z_{t}=\sigma \left(W_{z}x_{t}+U_{z}h_{t-1}\right),\;r_{t}=\sigma \left(W_{r}x_{t}+U_{r}h_{t-1}\right)$$

(15) $$\tilde{h_{t}}=\tanh\left(W_{h}x_{t}+U_{h}\left(r_{t}⊙h_{t-1}\right)\right),h_{t}=\left(1-z_{t}\right)⊙h_{t-1}+z_{t}⊙\tilde{h_{t}}$$

Here, $\sigma \left(\cdot \right)$ is the sigmoid function and ⊙ denotes the Hadamard product. The hidden state at the last time step $h_{T}$ is taken, with Dropout applied, as the global temporal feature:

(16) $$F_{gru}=\mathrm{Dropout}\left(h_{T}\right)$$

The outputs of the two branches are concatenated to form the fused representation:

(17) $$F_{concat}=\mathrm{Concat}\left(F_{cnn},F_{gru}\right)$$

The fused features are then passed through fully connected layers and Dropout to produce the single-neuron classification output:

(18) $$\hat{y}=\sigma \left(W_{2}\cdot \mathrm{Dropout}\left(\mathrm{ReLU}\left(W_{1}F_{\mathrm{cna}}+b_{1}\right)\right)+b_{2}\right)$$

where $\hat{y}\in \left(0,1\right)$ denotes the probability that the input sample belongs to the ADHD class, and $W_{1},{\;\;W}_{2},{\;b}_{1},{\;b}_{2}$ are trainable parameters.

#### 2.4.3. Performance Feedback-Driven Parameter Optimization Mechanism

**Figure 5:**
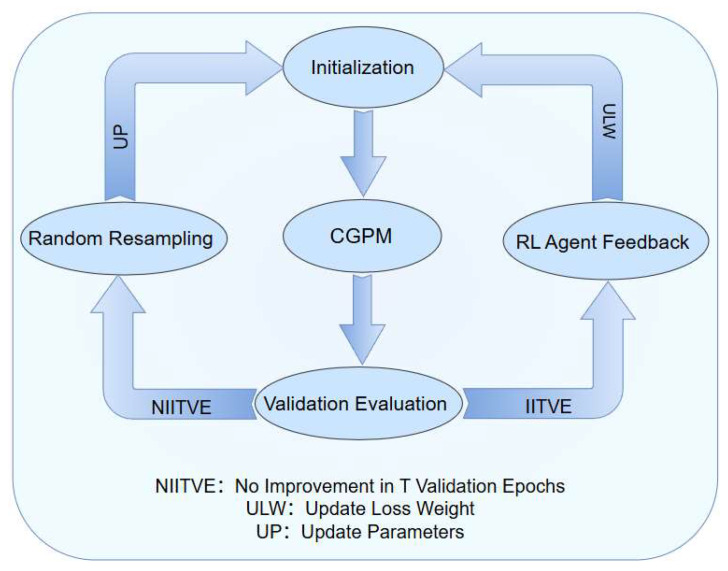
Framework of the PFPO mechanism.

This mechanism uses validation performance as feedback to dynamically adjust key training parameters, building a closed-loop adaptive optimization pathway to ensure convergence and robustness. The overall structure is shown in Figure 5.

At the end of each training epoch, a reward signal R is computed from validation performance:

(19) $$R={\mathrm{Val}}_{\mathrm{acc}}-{\mathrm{Val}}_{\mathrm{loss}}$$

where ${\mathrm{Val}}_{\mathrm{acc}}$ and ${\mathrm{Val}}_{\mathrm{loss}}$ denote validation accuracy and loss, respectively. A larger reward indicates higher accuracy and lower error. The loss weight factor w is then updated:

(20) w ←w + η⋅R

where η is the update step. A clipping function is applied to stabilize training:

(21) $$w=\mathrm{clip}\left(w,0.5,2.0\right)$$

This yields a weighted loss function for model optimization:

(22) $$L_{\mathrm{cso}}=w\cdot \mathrm{BCE}\left(y_{\mathrm{true}},y_{\mathrm{pred}}\right)$$

To prevent local optima or stagnation, hyperparameter resampling and network reinitialization are triggered when:

(23) $${\mathrm{Val}}_{\mathrm{acc}}\leq {\;\mathrm{Best}}_{val_{acc}}+\epsilon \;\;\mathrm{for}\;\;T\;\;\mathrm{consecutive}\;\mathrm{epochs}$$

where ϵ is the tolerance and T the stagnation threshold. New configurations are randomly sampled from the preset space:

(24) Dropout Rate∈{0.4,0.5,0.6,0.7}

(25) $$\mathrm{Learning}\;\mathrm{Rate}\in \{10^{-3},10^{-4},5\times 10^{-5}\}$$

(26) $$L2\;\mathrm{Regularization}\in \{10^{-3},5\times 10^{-4},10^{-4}\}$$

The model is then reinitialized with the new parameters, forming a fresh training start and breaking performance bottlenecks.

In addition, to enhance robustness, when weight updates fail to improve performance for T rounds, a perturbation reset is applied by resampling:

(27) $$w∼U\left(0.7,1.3\right)$$

This strategy approximates “policy perturbation” in reinforcement learning, helping to escape local optima and improving both diversity and stability in training.

Compared with traditional techniques such as early stopping and learning rate scheduling, the proposed PFPO mechanism builds a closed-loop adaptive optimization pathway driven by validation feedback. Instead of relying on fixed iteration counts or pre-set criteria, PFPO dynamically adjusts parameters based on real-time training performance, thereby enhancing convergence robustness and preventing premature stopping or inappropriate learning rate decay.

#### 2.4.4. Feature-Level Data Augmentation Strategy

During training, Gaussian noise and Cutout masking were introduced to impose perturbations and partial omissions on the input features, thereby improving model adaptability to fluctuations and missing data, as well as enhancing stability and robustness.

First, Gaussian noise is added to the original feature matrix $X\in \;R^{N\times T}$ to simulate background interference and electrode fluctuations during EEG acquisition:

(28) $$\tilde{X^{\mathrm{noise}}}=X+\epsilon ,\;\;\;\epsilon \;∼\;N\left(0,\;\sigma^{2}\right)$$

where ϵ is a Gaussian white noise matrix of the same size as X, with σ= 0.05. The noise intensity σ was selected based on empirical sensitivity tests across a range of small perturbation levels ( σ∈[0.01,0.1]). This value provided the most stable classification performance, offering an effective balance between feature diversification and signal fidelity.

Second, a random masking strategy (Cutout) was applied: for each sample $x_{i}\in {\;R}^{T}$, a segment of length L=0.1T starting at random s was set to zero:

(29) $$\tilde{x_{i}}\left(t\right)=\left\{\begin{array}{c} 0\;\;\;,\;t\in \left[s,s+L\right)\\ \tilde{x_{i}}\left(t\right),\;otherwise \end{array}\right.$$

This simulates local feature loss and encourages stronger modeling of global temporal structures.

On this basis, original, noise-augmented, and cutout-augmented samples were concatenated to build an extended dataset:

(30) $$X_{train}^{\mathrm{aug}}=\left[X,\tilde{X^{\mathrm{noise}}},\tilde{X^{\mathrm{cutout}}}\right],y_{train}^{\mathrm{aug}}=\left[y,y,y\right]$$

The enhanced training set expands the sample size from N to 3N, alleviating data sparsity and distribution bias, and improving the model’s ability to recognize ADHD-related EEG features.

## 3. Results

### 3.1. Experimental Setup

The experiments were conducted on a rented workstation equipped with an NVIDIA GeForce RTX 2080Ti GPU (NVIDIA), using the TensorFlow 2.x framework for model construction and training. To enhance sample diversity and generalization, the FDAS strategy was applied at the feature level, and the augmented data were used as model input.

The model adopts the CGPM structure, with training integrated with PFPO to dynamically adjust the dropout rate, L2 regularization, and learning rate based on validation performance, thereby improving convergence and robustness. Training employed the Adam optimizer with an initial learning rate of 1 × 10^−4^, a batch size of 64, and up to 500 epochs, combined with early stopping and learning rate scheduling to prevent overfitting.

All input features were standardized with Z-score normalization before training. Model evaluation used five-fold subject-independent cross-validation, ensuring no subject overlap between training and test sets. In each fold, 80% of the data was used for training and 20% for validation. Data augmentation, including Gaussian noise addition and Cutout masking, was applied only to the training samples to improve model generalization. After completing the five folds, the average performance (mean ± standard deviation) was calculated as the final result, ensuring a fair and reliable evaluation of the model’s generalization ability.

The proposed MSRLNet model is lightweight and computationally efficient, containing approximately 145,221 trainable parameters. Its estimated computational cost is around 0.29 MFLOPs per forward pass for an input of size 71 × 1, and the inference time per sample on the RTX 2080Ti GPU is roughly 3 milliseconds. These characteristics indicate that the model achieves a favorable balance between complexity and efficiency while maintaining high performance.

### 3.2. Evaluation Metrics

Model performance was evaluated using Accuracy, Cohen’s Kappa (κ), Precision, Recall, F1-score, and Root Mean Square Error (RMSE), with confusion matrices visualizing classification results.

Accuracy measures overall classification correctness, defined as:

(31) $$Accuracy=\frac{TP+TN}{TP+TN+FP+FN}$$

Precision and Recall represent the correctness and sensitivity of positive predictions, respectively, and F1-score is their harmonic mean:

(32) $$\mathrm{Precision}=\frac{TP}{TP+FP},\;\mathrm{Recall}=\frac{TP}{TP+FN},\;\;F1=\frac{2\cdot \mathrm{Precision}\cdot \mathrm{Recall}}{\mathrm{Precision}+\mathrm{Recall}}$$

Kappa quantifies overall agreement between predictions and true labels:

(33) $$\kappa =\frac{p_{o}-\;p_{e}}{1-{\;p}_{e}}$$

where $p_{o}$ is the observed agreement and $p_{e}$ the expected agreement.

RMSE evaluates the deviation between predicted probabilities and true labels:

(34) $$\mathrm{RMSE}=\sqrt{\frac{1}{n}\sum_{i=1}^{n}{\left(y_{i}-\hat{y_{i}}\right)}^{2}}$$

Here, TP, TN, FP, and FN denote true positives, true negatives, false positives, and false negatives, while $\hat{y_{i}}$ and $y_{i}$ are the predicted and true values of sample i. All metrics were computed across five-fold cross-validation and reported as mean ± standard deviation.

### 3.3. Overall Decoding Performance Analysis

The classification performance was compared across methods using key metrics such as Accuracy, F1-score, Precision, and Recall, along with visualizations including heatmaps and confusion matrices, to assess feature recognition ability and robustness in ADHD classification, thereby validating the comprehensive advantages of MSRLNet in accuracy, stability, and interpretability.

#### 3.3.1. Comparative Experiments

Table 2 presents the performance comparison of MSRLNet with several representative models on the same ADHD dataset. MSRLNet achieved or surpassed the best existing models in four key metrics—Accuracy (98.90%), F1 (98.98%), Precision (98.48%), and Recall (98.91%)—showing excellent individual identification capability. Compared with [40], Accuracy improved by about 22.8%, highlighting MSRLNet’s enhanced sensitivity to subtle brain dynamics. Against the recent deep model [41], MSRLNet gained 0.02% in Accuracy and 0.68% in F1, reflecting the marginal benefits of microstate feature fusion and adaptive parameter optimization.

In addition to the performance comparison presented in Table 2, we provide a methodological comparison of MSRLNet with representative ADHD classification models in Table 3. This table summarizes the core innovations, feature representations, and structural characteristics of each model. MSRLNet stands out by integrating dynamic microstate features with multi-dimensional statistical descriptors under a feedback-driven CNN–GRU framework, offering superior interpretability and robustness compared to existing approaches.

**Figure 6:**
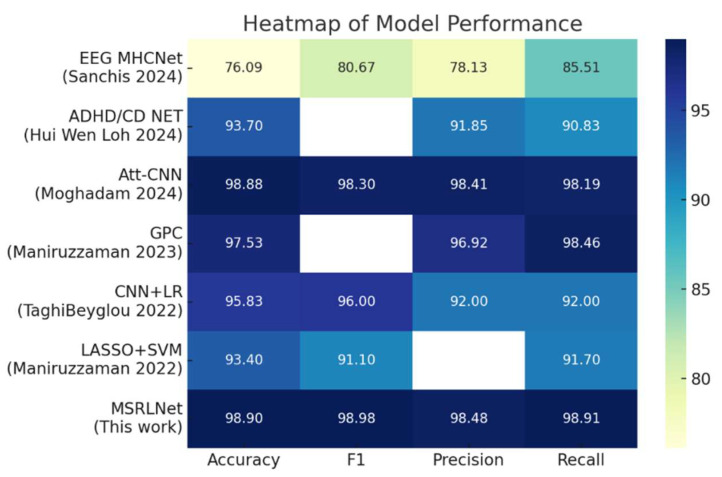
Performance Heatmap of Different Models across Four Evaluation Metrics [40,41,42,43,44,45].

The performance heatmap in Figure 6 clearly illustrates each model’s results across the four metrics, with MSRLNet showing the strongest intensity in all dimensions, indicating its overall advantage in classification accuracy and stability. Compared with traditional methods [43,44,45], MSRLNet achieves better balance between Precision and Recall, underscoring its strength in modeling non-stationary signals and clinical adaptability.

Overall, the comparative experiments further confirm the rationality and necessity of the proposed modular design, providing solid support for ADHD EEG feature recognition.

#### 3.3.2. Subject-Independent Analysis

To evaluate the classification performance and generalization of MSRLNet in ADHD EEG feature recognition, a subject-independent five-fold cross-validation was applied to systematically test model stability and robustness. Results (Table 4) show all metrics above 98%, Kappa close to 1, and RMSE below 0.11, indicating that the model achieves high-accuracy prediction and robust generalization even under sparse samples and class imbalance.

### 3.4. Ablation Study

To assess the contribution of each module to MSRLNet, we conducted systematic ablation experiments (Table 5). The results show:

1. MSFF (feature fusion module) is essential: Removing MSFF reduced accuracy from 98.9% to 77.6–88.7%, indicating that fusing microstate and statistical features is irreplaceable in ADHD recognition.
2. PFPO (feedback optimization) enhances robustness: Without PFPO, accuracy remained around 96.6%, but generalization and training stability declined, confirming its value for handling non-stationary signals and optimizing convergence.
3. FDAS (data augmentation) mitigates overfitting: Excluding FDAS led to a clear drop in recall, showing its benefit in improving recognition of limited and boundary samples.
4. CGPM (CNN–GRU parallel structure) is the backbone: CGPM, as the core of temporal modeling, was retained in all settings; its synergy with other modules ensures multi-scale dynamic modeling capability.

In summary, MSFF, PFPO, and FDAS each play key roles, and combined with CGPM enable MSRLNet to reach optimal performance. The ablation results further demonstrate that the integration of microstate and statistical features provides complementary and non-redundant information, significantly enhancing both accuracy and interpretability compared with single-source feature modeling. This further validates the unique advantages of microstate feature fusion and feedback-driven parameter tuning in ADHD EEG recognition.

### 3.5. Key and Mechanistic Role of Microstate Features in ADHD EEG Recognition

Ablation results show that MSFF provides significant gains in MSRLNet. This section analyzes the discriminative contribution of microstates from both performance comparison and neural interpretation.

**Figure 7:**
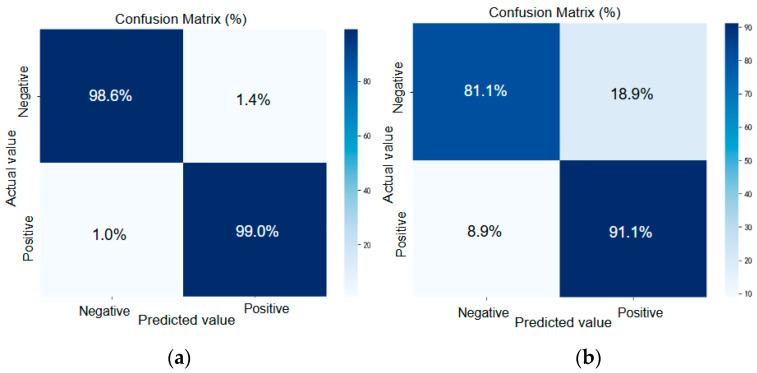
Comparison of average confusion matrices on the test set. (**a**) Full model. (**b**) Without microstate features.

As shown in Figure 7, the full model with microstate features (Figure 7a) achieved accuracies of 98.7% for ADHD and 98.4% for controls, whereas removing this module (Figure 7b) reduced accuracy to 92.7% and 84.0%, with misclassification notably increased. This demonstrates the key role of microstate features in distinguishing EEG patterns.

**Figure 8:**
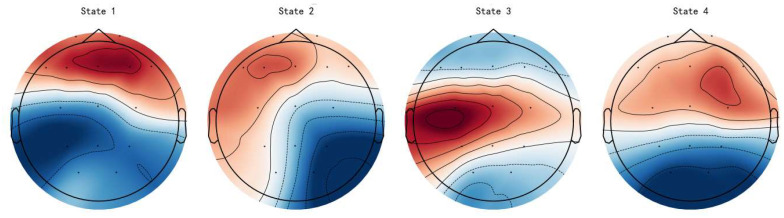
Topographiesof the four EEG microstates obtained from our dataset. Red indicates positive scalp potentials, while blue indicates negative scalp potentials.

Microstates segment whole-brain EEG into transient spatial configurations. The four common classes (A, B, C, D) correspond to distinct functional networks (Figure 8): A with the bilateral temporal language network, reflecting stable or initial cognition; B with the right frontoparietal network, related to alertness and attention regulation; C with the posterior default mode network (pDMN), involved in introspection and cognitive integration; and D with the medial frontal and prefrontal networks, engaged in executive control and impulse inhibition.

The group comparison of microstate features not only highlights the improved discriminative performance of the model but also reveals systematic alterations in the EEG dynamics of individuals with ADHD.

As shown in the transition path analysis (Table 6), ADHD subjects exhibit significant deviations in several key transitions. Specifically, the probabilities of A → B and D → B are significantly increased, suggesting a greater tendency for brain activity to shift from initial or high-arousal states into the activation state, reflecting impulsive initiation and heightened arousal. In contrast, the probabilities of A → C, D → C, and B → C are markedly reduced, indicating impaired efficiency in transitioning from initial or activation states to the integration state, and difficulty in achieving stable cognitive regulation and resource coordination. Overall, the EEG dynamics of ADHD show an asymmetric pattern of “excess activation transitions with reduced integration,” consistent with clinical features of impaired attention maintenance and poor impulse control.

In the comparison of mean duration (Table 7), the B microstate of ADHD subjects was significantly prolonged, whereas the C microstate was markedly shortened. This indicates that ADHD individuals remain longer in activation-dominant states but show reduced stability in integration and cognitive control states.

The time coverage results (Table 8) revealed a consistent pattern. The coverage of the B microstate was significantly increased in ADHD subjects, suggesting that their overall brain activity is more dominated by activation states; in contrast, the coverage of the C microstate was significantly reduced, indicating insufficient temporal allocation to networks related to integration and cognitive control. This macroscopic shift in coverage, consistent with duration differences, reflects a systemic imbalance in the EEG dynamics of ADHD.

**Figure 9:**
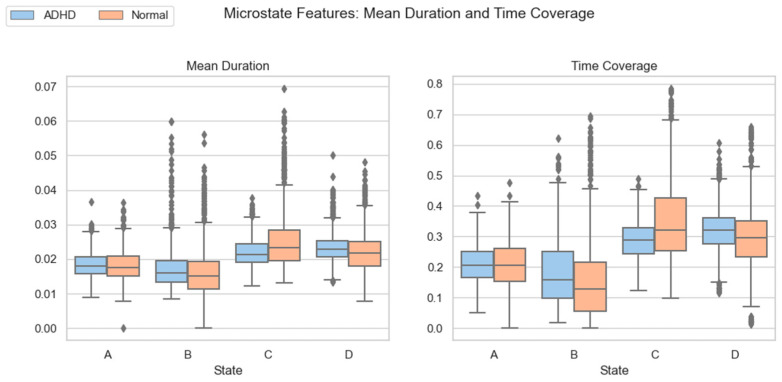
Group comparison of microstate mean duration and time coverage. Letters A–D represent microstates A, B, C, and D, respectively.

To provide an intuitive view of group differences, Figure 9 presents boxplots of the mean duration and coverage for the four microstates. The plots clearly show that ADHD subjects score higher than controls on B-related indices but significantly lower on C-related indices, further underscoring the imbalance between activation and integration states.

In summary, ADHD individuals exhibit a systemic shift in microstate dynamics: their brain activity tends to remain more frequently and persistently in the activation state B, while transitions and resource allocation to the integration state C are markedly reduced. This pattern not only reflects their clinical manifestations of attentional deficits and heightened impulsivity but also confirms the value of microstate features in revealing EEG dynamical abnormalities in ADHD and providing discriminative biomarkers.

## 4. Discussion

Systematic experiments validated the effectiveness and superiority of the proposed MSRLNet in ADHD EEG feature recognition. Results show that MSRLNet significantly outperforms comparative methods in key metrics such as Accuracy, F1-score, and Kappa, while maintaining stable performance across folds, reflecting strong robustness. This confirms the rationality and efficiency of the multi-source feedback modeling approach.

At the structural level, the CGPM captures both local spatial features and long-range temporal dependencies within one framework, overcoming the limitations of single-path models. The PFPO enables adaptive control during training, ensuring good convergence and stability even on small datasets. FDAS further enriches feature distribution diversity, effectively mitigating overfitting and enhancing generalization.

At the feature level, introducing microstate features provides crucial support for ADHD EEG recognition. Previous studies show that microstates reflect brain functional state switching at the millisecond scale, and ADHD individuals exhibit significant alterations in these spatiotemporal patterns. Our experiments confirm this: removing microstate features caused a marked drop in classification performance, and T-SNE visualization showed class boundaries becoming overlapped and blurred, further highlighting their importance in ADHD EEG recognition. “These results provide clear evidence that the extracted features are highly distinguishable between ADHD and Normal groups, directly supporting the validity of our feature design”.

**Figure 10:**
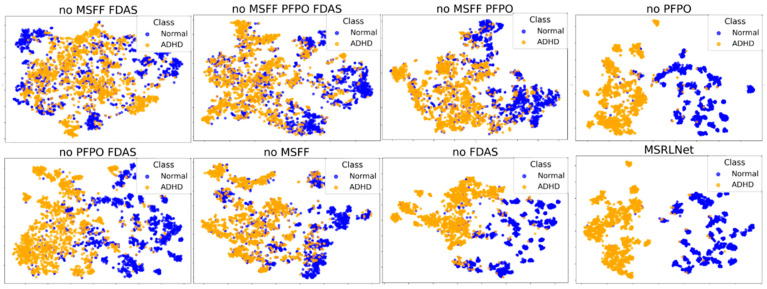
t-SNE visualization demonstrating the effectiveness and necessity of each module in the model.

The T-SNE results also offer intuitive evidence for model interpretability. As shown in Figure 10, the full MSRLNet forms compact and well-separated clusters in feature space, whereas removing modules such as MSFF, PFPO, or FDAS led to reduced inter-class distance and blurred boundaries. “These findings highlight the contribution of each module in enhancing feature separability and stability, demonstrating their critical role in improving model robustness”.

From a clinical perspective, the contribution of MSRLNet lies not only in performance gains but also in its potential clinical value. By integrating microstate features, the model uncovers abnormal patterns in the dynamic organization of brain function in ADHD, offering a new lens to understand its neural mechanisms. Such interpretability holds promise for aiding diagnosis, monitoring interventions, and guiding personalized treatment.

“Beyond performance metrics, MSRLNet also achieves a favorable balance between computational cost and interpretability. The feedback-driven optimization mechanism allows efficient convergence and reduced training overhead, while maintaining stable accuracy across folds. Meanwhile, the inclusion of microstate features enhances neurophysiological interpretability, linking network decisions to specific alterations in cognitive state transitions. These aspects collectively strengthen the translational potential of MSRLNet from research settings to real-world clinical applications”.

Nevertheless, this study has limitations. First, the dataset is relatively small. Although data augmentation and cross-fold validation helped reduce overfitting, the model’s generalizability needs further testing on larger, multi-center datasets. “In addition, our current results were obtained from a single-center dataset under a unified EEG acquisition protocol. Therefore, the model’s performance may vary when applied to data collected from different centers or using different EEG systems, electrode configurations, or sampling rates. Evaluating MSRLNet on multicenter or cross-protocol datasets in future studies would help assess its robustness and external validity. Second, this work is based mainly on resting-state EEG, while ADHD-related functional abnormalities may be more evident under task conditions. Future studies with task-based EEG or longitudinal data would be more valuable”.

In conclusion, MSRLNet shows marked performance advantages and strong interpretability in ADHD EEG recognition, providing a new path for building efficient and reliable EEG analysis models, with important potential in clinical support.

## 5. Conclusions

This study proposes a multi-source feedback network (MSRLNet) that integrates EEG microstates and statistical features to improve the accuracy and interpretability of ADHD recognition. MSRLNet combines CGPM, PFPO, and FDAS. This design enhances robustness while fully capturing multi-scale EEG dynamics and microstate topologies. Experimental results show that MSRLNet outperforms baseline methods on key metrics and maintains high stability in cross-fold validation. Ablation studies further confirm the necessity of each module, highlighting that microstate features not only boost classification but also strengthen neurophysiological interpretability.

Compared with models relying only on time- or frequency-domain features, MSRLNet shows three key advantages: (1) by integrating microstate features, it effectively captures abnormal spatiotemporal patterns in ADHD; (2) the CGPM structure jointly models local spatial features and long-range temporal dependencies, avoiding the limits of single-branch designs; (3) PFPO and data augmentation reduce overfitting risks under small-sample conditions, greatly improving generalization. These findings indicate that MSRLNet not only achieves superior performance but also provides a new modeling paradigm for objective ADHD diagnosis.

Future work can expand in several directions: validating on multi-center datasets to assess generalizability across settings and populations; exploring cross-modal fusion (e.g., combining EEG with fNIRS or fMRI) to better characterize ADHD brain dysfunction; introducing attention mechanisms or graph neural networks (GNNs) to refine spatiotemporal and topological modeling of EEG; incorporating longitudinal and task-related data to probe ADHD dynamics across development and cognitive states; and, clinically, deploying MSRLNet as a real-time EEG screening tool enhanced with explainable AI to improve interpretability and physician trust.

In summary, MSRLNet demonstrates strong performance and interpretability in ADHD EEG recognition, with promising potential for clinical diagnosis and personalized intervention. “However, since this study was conducted on a single-center dataset with a limited sample size, the generalization of the proposed framework should be interpreted with caution. Future research should focus on replicating these findings across diverse datasets and real-world clinical environments to further confirm its robustness and applicability”.

## Figures and Tables

**Table 1:** Extracted 40 Features in 26 Categories from EEG Data.

Extracted Features	Extracted Features
Standard deviation	Hjorth mobility [24]
Peak-to-peak amplitude	Hjorth complexity [24]
Skewness	Higuchi fractal dimension [24,25]
Kurtosis	Katz fractal dimension [25]
Root-mean squared value	Number of zero-crossings
Quantile	Line length [26]
Hurst exponent [27,28]	Spectral slope [29,30]
Approximate entropy [31]	Spectral Entropy [32]
Samp entropy [31]	SVD entropy [33]
Decorrelation time [34]	SVD Fisher Info [33]
Pow Freq Bands [34]	Energy Freq Bands [35]
Hjorth mobility from power spectrum [34,36]	Spectral Edge Frequency [36]
Hjorth complexity from power spectrum [34,36]	Db4 Wavelet energy (x6 levels of decomposition) [34]

**Table 2:** Horizontal Comparison with Existing Models.

Author (Year)	Model Name	Accuracy (%)	F1	Precision	Recall
Sanchis J (2024) [40]	EEG-MHCNet	76.09%	80.67%	78.13%	85.51%
Loh H W (2024) [42]	ADHD/CD-NET	93.70%	—	91.85%	90.83%
Ahmadi Moghadam E (2024) [41]	Att-CNN	98.88%	98.30%	98.41%	98.19%
Maniruzzaman M (2023) [43]	GPC	97.53%	—	96.92%	98.46%
TaghiBeyglou B (2022) [44]	CNN + LR	95.83%	96%	92%	92%
Md. Maniruzzaman (2022) [45]	LASSO + SVM	93.40%	91.10%	—	91.70%
This work	MSRLNet	**98.90%**	**98.98%**	**98.48%**	**98.91%**

**Table 3:** Methodological Comparison of MSRLNet and Representative ADHD Classification Models.

Model Name	Core Innovation (Including Structure)	Feature Representation	Advantage of MSRLNet
EEG-MHCNet [40]	Multi-head CNN for channel optimization	Channel subset features	Significant improvement in interpretability and performance
ADHD/CD-NET [42]	Explainable deep CNN with Grad-CAM visualization	CWT correlation matrices	MSRLNet achieves higher generalization and stability
Att-CNN [41]	Attention CNN combining linear (PCC) and nonlinear (PLV) connectivity	Connectivity fusion maps	MSRLNet adds adaptive feedback and dynamic modeling
GPC [43]	Gaussian Process Classifier with channel and feature selection	28 handcrafted static EEG features	Deep learning improves dynamics and nonlinearity
CNN + LR [44]	Hybrid CNN + Logistic Regression model	Morphological and spectral EEG features	Multi-domain fusion yields better robustness
LASSO + SVM [45]	LASSO feature selection + SVM (RBF) classifier	Morphological + time-domain descriptors	Dynamic microstate modeling captures richer temporal info
MSRLNet	**Multi-source feedback-driven CNN–GRU framework (CGPM + PFPO); integrates dynamic and static features for interpretable ADHD recognition**	**EEG microstate features + statistical descriptors + FDAS**	**—** **(Proposed model)**

**Table 4:** Performance of MSRLNet in five-fold cross-validation.

Metric	Mean	RMSE
Accuracy	98.90%	±0.54
Kappa	0.979	±0.008
F1-score	98.98%	±0.53
Precision	98.48%	±0.71
Recall	98.91%	±0.66
RMSE	0.104	±0.007

**Table 5:** Ablation Study Results: Impact of Each Module on MSRLNet Performance.

Model No.	MSFF	PFPO	CGPM	FDAS	Accuracy (%)	Kappa	F1 (%)	Precision (%)	Recall (%)
1	×	√	√	√	88.70	0.866	89.88	87.25	92.68
2	√	×	√	√	96.56	0.959	97.09	98.46	96.78
3	√	√	√	×	94.82	0.895	95.27	94.19	95.37
4	×	×	√	√	84.19	0.678	86.21	81.77	91.18
5	×	√	√	×	82.33	0.639	84.81	79.45	91.04
6	√	×	√	×	89.40	0.784	90.67	86.71	94.89
7	×	×	√	×	77.56	0.541	80.76	75.39	86.96
8	√	√	√	√	98.90	0.979	98.98	98.48	98.91

**Table 6:** Analysis of microstate transition path differences (↑ indicates an increase, while ↓ indicates a decrease).

Path	ADHD ↑/↓	Significance	Neural Implication
A → B	↑ (sig.)	*p* < 0.05	Stronger activation initiation, weaker control
D → B	↑ (sig.)	*p* < 0.05	Frequent high-arousal cycles, hard to interrupt
A → C	↓ (sig.)	*p* < 0.05	Impaired integration, weak regulation
D → C	↓ (sig.)	*p* < 0.05	Difficulty shifting to integration state
B → C	↓ (sig.)	*p* < 0.05	Missing convergence, sustained attention deficit

**Table 7:** Differences in average microstate duration (↑ indicates an increase, while ↓ indicates a decrease).

Microstate	ADHD ↑/↓	Significance	Neural Implication
B	↑ (sig.)	*p* < 0.05	Activation state lasts longer
C	↓ (sig.)	*p* < 0.05	Integration state unstable

**Table 8:** Differences in microstate time coverage (↑ indicates an increase, while ↓ indicates a decrease).

Microstate	ADHD ↑/↓	Significance	Neural Implication
B	↑ (sig.)	*p* < 0.05	Brain activity biased to activation
C	↓ (sig.)	*p* < 0.05	Weakened integration/cognitive control

## Data Availability

The data supporting the findings of this study are openly available at IEEE Dataport under the title *EEG Data for ADHD and Control Children*, accessible at https://ieee-dataport.org/open-access/eeg-data-adhd-control-children.
